# Supplementary figures and images for: Generating information-dense promoter sequences with optimal string packing
Source: PLoS Comput Biol. 2024 Jul 24;20(7):e1012276. doi: 10.1371/journal.pcbi.1012276 (PMC11268586; doi:10.1371/journal.pcbi.1012276)

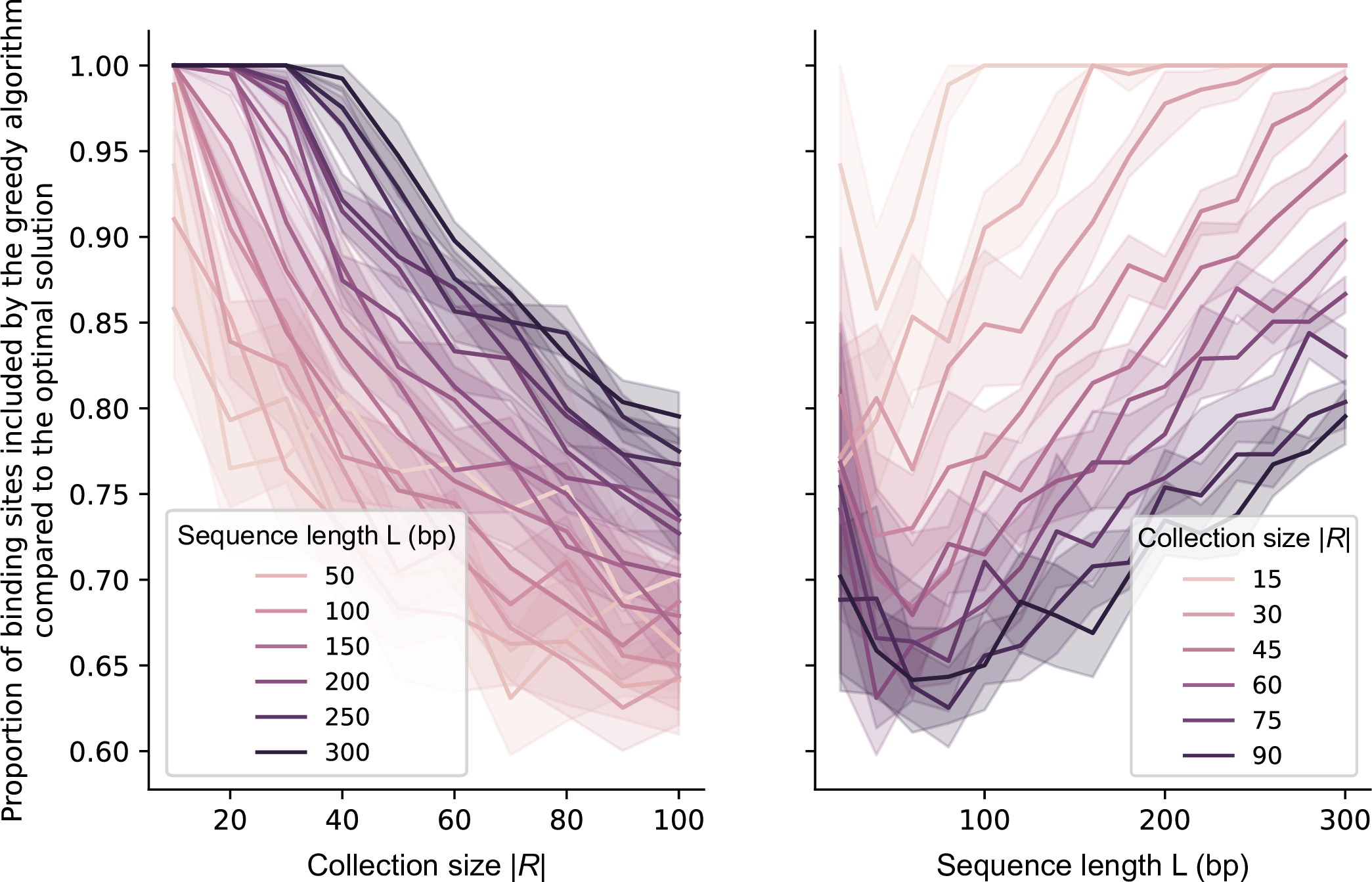

Supplement: S1 Fig — We plotted the number of binding sites that the approximation algorithm managed to fit into a sequence of a given length, L, normalized by the optimal solution. We repeated each scenario 10 times with random binding sites of uniform random lengths between 5 and 15 base pairs. Shaded regions represent the bootstrapped 95% confidence interval around the mean. (TIF) [file pcbi.1012276.s001.tif]

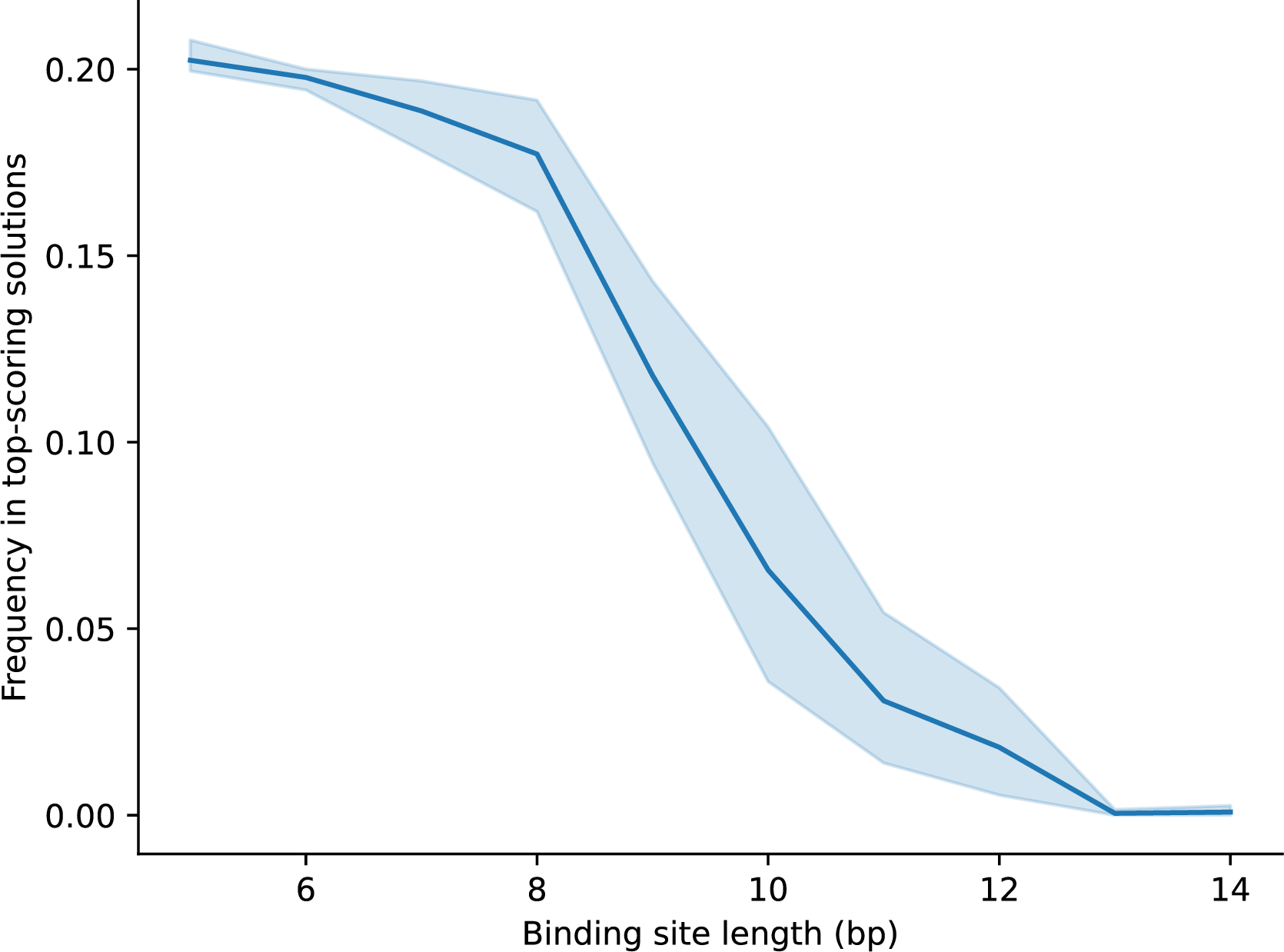

Supplement: S2 Fig — Ten binding site collections were randomly generated with 10 binding sites each, where there is one binding site of each length from 5 to 14 base pairs. All of the top-scoring solutions for a sequence length L = 50 were generated every time: for one of the 10 binding site collections, 4 binding sites were able to be fit at best, with 7184 ways to do so. For the nine others, 5 binding sites were able to be fit at best, with 26, 72, 150, 184, 206, 278, 418, 664, 762 ways to do so. The shaded region represents the bootstrapped 95% confidence interval around the mean. (TIF) [file pcbi.1012276.s002.tif]

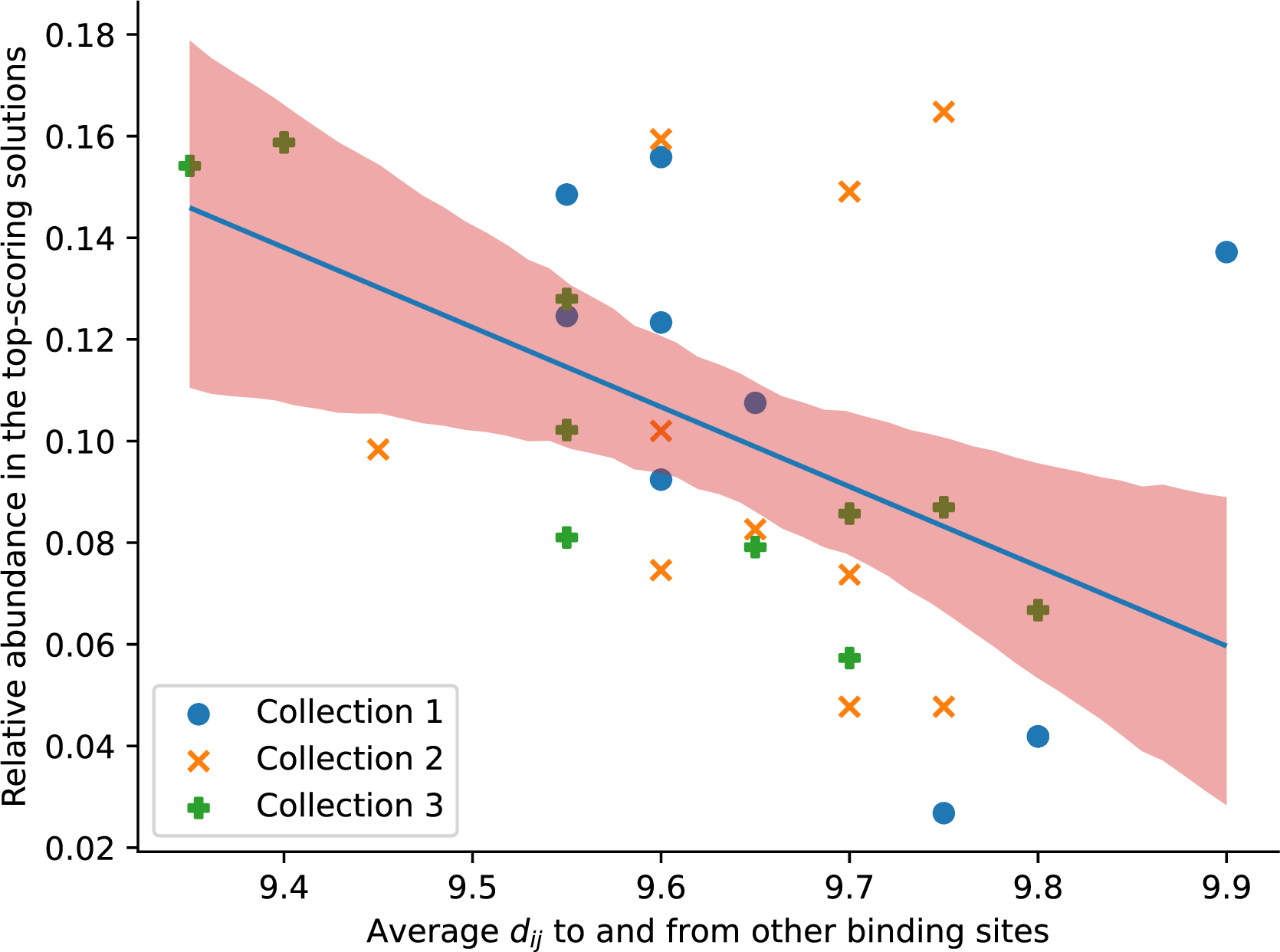

Supplement: S3 Fig — The blue line is the average linear regression line, the shaded region is the 94% credible interval of the mean. (TIF) [file pcbi.1012276.s003.tif]

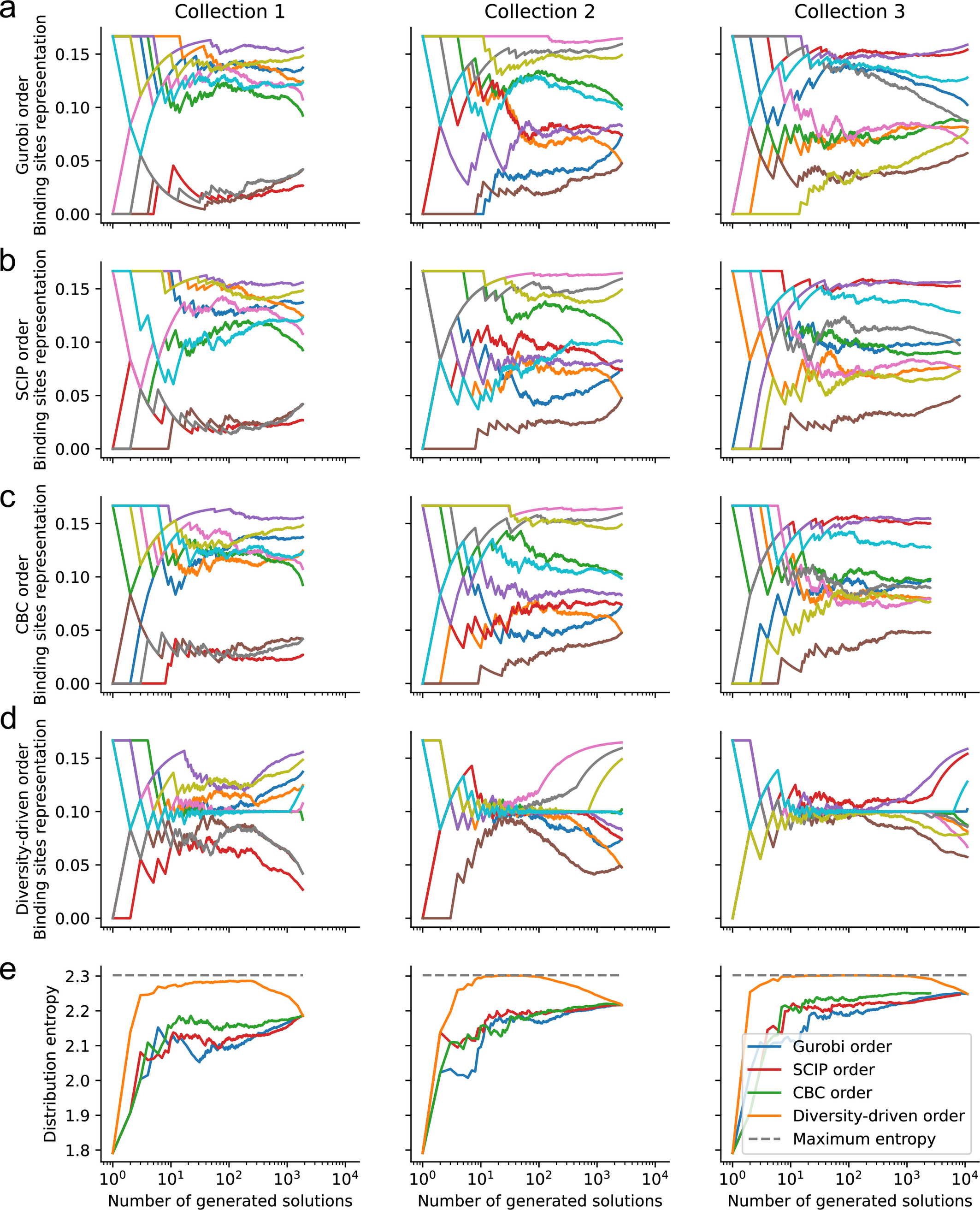

Supplement: S4 Fig — Solver order with (A) Gurobi, (B) SCIP, and (C) CBC solvers. (D) Diversity-driven order control strategies. Details on the solve times for the different solvers are available in S1 Table. (E) The diversity-driven order approach generally produces higher entropy distributions despite the bias in binding site representation. Note that data from Fig 4 for the Gurobi solver, diversity-driven order, and entropy plots are replicated here for ease of comparison. (TIF) [file pcbi.1012276.s004.tif]

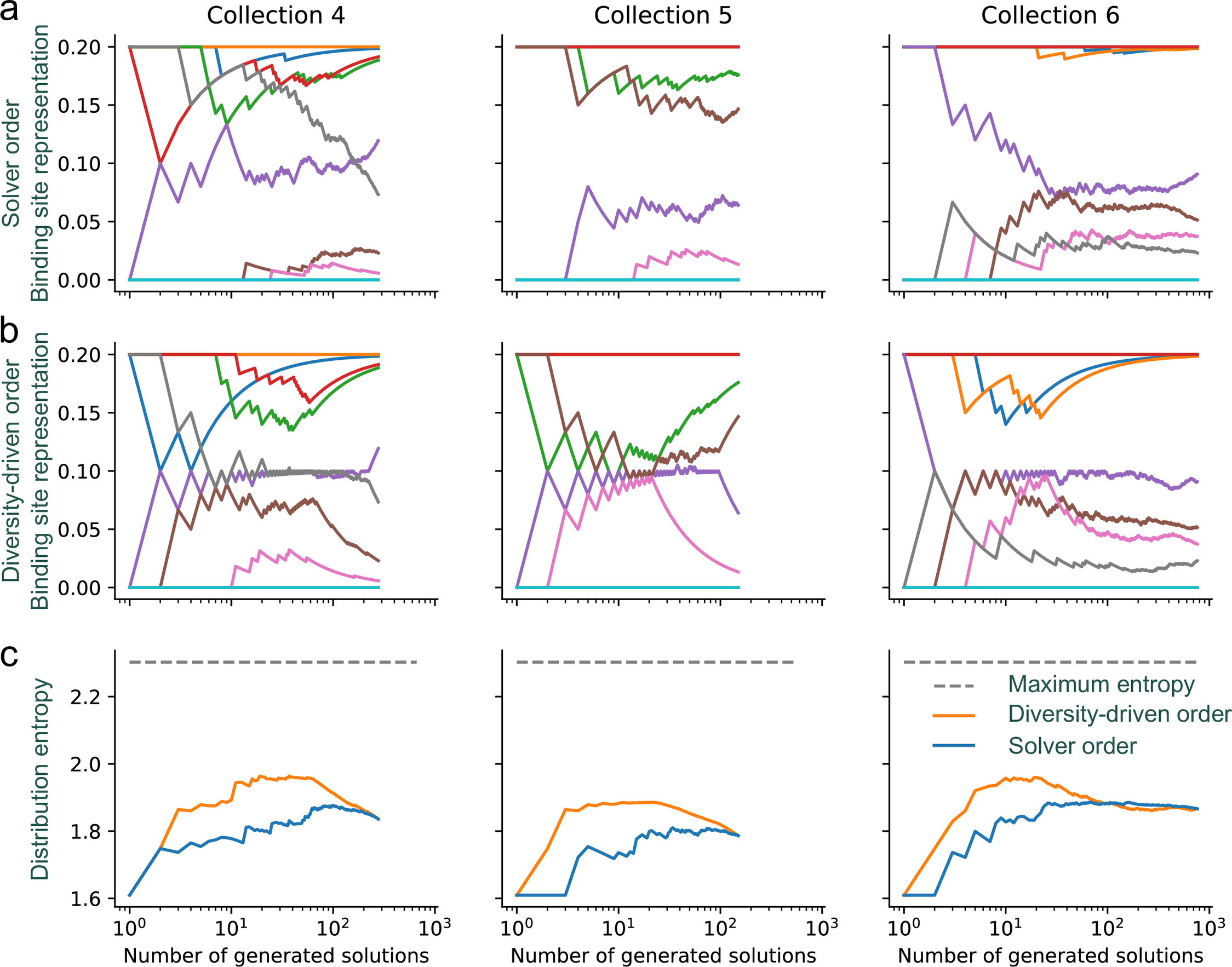

Supplement: S5 Fig — The solver order (A) and diversity-driven order (B) strategies applied to heavily biased libraries, where binding sites have different sizes. Every library is made of 10 randomly generated binding sites, one of size 5 base pairs, one of size 6, etc., until size 14. As the full distribution attests (rightmost point of the graphs), some binding sites are present in almost all top-scoring solutions, while some others are present in none. Despite this, the diversity-driven order approach generally produces higher entropy distributions (C). The solver used here was Gurobi. (TIF) [file pcbi.1012276.s005.tif]
