# Supplementary material for: Generating information-dense promoter sequences with optimal string packing
Source: PLoS Comput Biol. 2024 Jul 24;20(7):e1012276. doi: 10.1371/journal.pcbi.1012276 (PMC11268586; doi:10.1371/journal.pcbi.1012276)
Supplement: S1 Table — For solve times > 20 h, we stopped computation at this point. (PDF) [file pcbi.1012276.s006.pdf]

Supplementary Table

Table S1: Time to enumerate all top-scoring solutions using solver order, for three different solvers. For solve times > 20 h, we stopped computation at this point.

**Time to solve**	**Gurobi**	**SCIP**	**CBC**
**Collection 1 (1854 solutions)**	11 m 20 s	2 h 46 m 46 s	16 h 02 m 48 s
**Collection 2 (2654 solutions)**	22 m 04 s	6 h 19 m 44 s	> 20 h (2631 sols in 20 h)
**Collection 3 (10922 solutions)**	3 h 29 m 28 s	> 20 h (7997 sols in 20 h)	> 20 h (2498 sols in 20 h)
