# Supplementary material for: Generating information-dense promoter sequences with optimal string packing
Source: PLoS Comput Biol. 2024 Jul 24;20(7):e1012276. doi: 10.1371/journal.pcbi.1012276 (PMC11268586; doi:10.1371/journal.pcbi.1012276)
Supplement: Attachment — Submitted filename: Response to Reviewers.pdf [file pcbi.1012276.s007.pdf]

Point-by-Point Response to Reviewers
Manuscript Number: PCOMPBIOL-D-24-00208

We appreciate the reviewers' comments and suggestions for improving the content and the clarity of the manuscript. We have addressed their concerns in the revised manuscript. A complete list of changes and detailed responses to the comments follows.

Reviewer 1:

In the manuscript entitled "Generating information-dense promoter sequences with optimal string packing," the authors described solution methods to create promoter sequences that contain many transcription factor binding sites in a specified (typically short) length of DNA. The final solver developed for this task is available online, and the ability to generate promoters with densely packed binding sites could be of general interest to the synthetic biology or cell engineering communities. However, the functionality of at least one promoter library must be shown to demonstrate the expected value of this novel solution method. Demonstration of bacterial promoter library function would suffice.

> We wholeheartedly agree that experimental validation is important. However, generating a well-designed promoter library is not a trivial task and is a substantial undertaking in its own right. This is something that we have recently started to work on, but it will be a manuscript-scale set of results, which is why we chose to break this work into two parts, one with a computational focus (this manuscript at PLOS Computational Biology), and in the future we anticipate writing a second experimentally-focused manuscript that we will submit to another journal.
>
> In principle, conducting a functional screen of promoter variants is straightforward. However, the SPP inherently designs nucleotide sequences with complex DNA-protein interactions, and this added sequence complexity introduces additional criteria for proper functional screening. These criteria involve selecting appropriate binding sites for inclusion in promoter variants, and designing assays that involve cellular events affecting multiple transcription factors. The structure of bacterial promoters is well-defined, with the strength of core promoters being primarily driven by the presence of sigma factor recognition sites. Therefore, in the right environmental context where the target sigma factor is active, it would likely be possible to design a small functional library and it would not be surprising for dense arrays that contain these well-known consensus sites to act as promoters. However, we are concerned that designing and presenting results from these "conventional," albeit synthetic, promoters would not convincingly showcase the strength of our method. Instead, the strength of the SPP lies in its ability to efficiently design sequences that accommodate complex, overlapping DNA-protein interactions. Furthermore, the SPP can generate these types of sequences at a scale that can facilitate studies on transcription factor signal integration, competition, and condition-dependent interactions influenced by genetic context. Studies of this complexity can require libraries ranging from tens-to-hundreds of thousands of variants.

The SPP itself is a considerable contribution to *in silico* biological sequence design. Existing studies that have attempted the design of overlapping sequences have relied on *ad hoc* methods or very short sequences, neither of which are appropriate for large-scale library design. There is a growing interest in designing synthetic DNA (i.e., sequences not found in natural genomes) both to develop parts with novel function and to generate training examples for machine learning models. At its core, the SPP offers a novel, scalable method for generating synthetic DNA sequences. We now describe this in further detail in the manuscript.

In summary, given the complexity of conducting a functional screen and the depth with which the current manuscript explores the SPP, we aim to maintain the focus here on the computational and application-agnostic aspects of the SPP, setting aside functional screening for more targeted future investigations.

→ See Introduction and Discussion

1. It is often unclear what the library size is for each generated library, referring to the number of sequences that would need to be screened to test for function (number of sequences generated), instead of the author's definition of library size [R] = number of binding sites. This information is necessary to gauge the usefulness of each generated library, as screening 10^6 promoters for function might be possible in one system, while testing 10^1 might be more feasible in another. As the manuscript sells the SSP method for promoter library generation, discussion of the feasibility of testing the generated libraries is warranted.

   We thank the reviewer for raising this important point, which was a potential source of confusion. We acknowledge that "library size" conventionally refers to the number of variants in a functional screening context, whereas we were using it to mean this but also to describe the binding site collection size $|R|$. To eliminate this source of confusion, we have revised the terminology throughout the manuscript. Now, "library" refers to the sequences generated for screening, and "binding site collection" describes the number of binding sites input into the SPP to generate a dense array.

   → See points throughout manuscript

   To the reviewer's second point, we agree that different promoter design projects may require varying library sizes, depending on the complexity of the desired expression profiles. In the context of integer linear programming and the SPP, the goal of generating sequences with specific characteristics—such as a defined spacing between two particular binding sites to achieve a desired promoter "likeness"—often translates into layering incentives or constraints onto the solver. This approach helps steer the types of solutions that are returned. We have observed that solver time increases as more constraints are applied. For example, tasks like generating dense arrays constrained only by binding site collection size $|R|$ and sequence length L (as in Figure 2) tend to be quicker. However, more complex generative tasks, such as creating dense arrays with "diversity-driven order" (Figure 4) or "positional bias" (Figure 6), add complexity and extend solve time. We have updated the Discussion to explicitly address this trade-off between speed and constraints.

We also include actual solve times for a representative library design task, comparing results across solvers.

→ See Discussion
→ See Table S1

2. Promoter library sequences, the full sequences in addition to the inputted binding sites, should be included in the supplement or extended data, primarily for libraries that are expected to have function like the bacterial promoter libraries.

To clarify, all figures utilized mock binding sites (i.e., randomly generated sequences representing binding sites). This decision was made to highlight the SPP's capabilities in an application-agnostic manner. We have now clearly stated in the Results section and figure captions that all binding sites were random sequences to avoid future confusion.

→ See points throughout the manuscript

Because readers may benefit from access to the data shown in the manuscript, all sequences described in the manuscript are available in our open-source "dense-arrays" library, linked in the Source Code section. The /benchmarks folder provides detailed information for each dense array. It specifies which binding sites from a given binding site collection (input into the SPP) appeared in the solution sequence, along with their coordinates represented as string offset positions. To enhance accessibility, we have explicitly stated in the manuscript that these sequences are available. Additionally, we have included a README file in the /benchmarks folder to help interpret the provided tables. The Source Code section now includes a description of how to access the data.

→ See Source Code

Reviewer 2:

Natural promoter regions may contain many overlapping binding sites of transcriptional factors, affecting transcription initiation rates. Despite the common occurrence of overlapping binding sites in nature, the rapid artificial design of nucleotide sequences with many overlapping sites remains a challenge. In this paper, the authors propose a computational approach for designing nucleotide sequences with densely packed DNA-protein binding sites, termed the Nucleotide String Packing Problem (SPP). They first demonstrate that the SPP problem is NP-hard, and thus simplify the problem into Orienteering Problem with integer distances, which can then be efficiently solved using various open-source and commercial solvers. The authors subsequently explore many possibilities of the method in the design of bacterial promoters.

1. Regarding the issue of bias in solutions, the authors attempt to explore the effects of binding site size and sequence on bias, while briefly mentioning the potential impact of different solvers due to their different internal algorithms. However, the explanation for the effects of

binding site sequences and different solvers is not sufficiently clear. For the effect of sequences, one approach could be to investigate the influence of bias from the perspective of sequence overlap. Additionally, exploring different solvers and observing their specific effects on bias, if any, could also be attempted here.

We thank the reviewer for these suggestions. We have now included more analysis on sequence bias, examined sequence overlap, and tested the effects of bias with different solvers.

Working with the binding site collections presented in Figure 3, we investigated the reasons for discrepancies in the representation of binding sites, which were all the same length, across various dense arrays. We found that binding sites whose sequence was more subject to overlap with other binding sites were the ones that were privileged in the top-scoring solutions (Figure S3).

→ See Results "Diversity of the generated solutions" section
→ See Figure S3

As for comparing the effect of different solver implementations on the transient bias in binding site representation among similarly-scored solutions, we updated Figure S4 to show that different solvers (Gurobi, SCIP, CBC) indeed return similarly-scored solutions in different arbitrary orders. None of the solvers maximize the entropy of binding site representation in transient solution sets. Additionally, we now include Table S1, which details the solve times for Gurobi, SCIP, and CBC, offering a practical perspective on performance relative to Figure S4.

→ See Results "Diversity of the generated solutions" section
→ See Figure S4
→ See Table S1

2. The article mentions that "Meanwhile, generative AI techniques are starting to show promise in emulating the complexity of context-dependent promoters (31–37). However, these models often struggle with interpretability, and fine-tuning them to include or exclude specific binding sites still requires specialized expertise (38)." However, in practice, the method designed in this article may rely more heavily on specialized expertise, as understanding different binding sites may involve complex processes. Additionally, whether existing expert knowledge is sufficient to generate binding site libraries consistent with natural promoters is worth discussing. It is recommended that the authors provide a clearer explanation in this regard.

We thank the reviewer for pointing this out, and indeed, contrasting our SPP approach with generative deep learning models, based on required user expertise, may not be an appropriate angle.

As an alternative, it is worth noting that vast majority of generative promoter models learn on large training sets of natural (non-synthetic) sequence examples (DOIs: 10.1038/s41467-023-41899-y; 10.1021/acssynbio.4c00041; 10.48550/arXiv.2402.06079).

Consequently, all generated promoters are derived from these natural sequences, and these models may struggle to create sequences outside their training distributions. The choice of training data crucially shapes the model, reflecting our assumptions about which sequence distributions we intend to explore (DOI: 10.1038/s41587-023-02115-w). The SPP can generate DNA sequences with "extreme" cis-regulatory logic that likely do not appear in the host's genome, offering the potential to present novel expression responses not dictated by the cell's evolutionary history.

To address these points, we have updated the Introduction to describe this distinction.

→ See Introduction

3. Furthermore, due to the ambiguity in determining binding sites in biology and the variation in protein-motif binding across different biological states, whether more densely distributed binding sites correspond to a more suitable promoter is still worth considering. It is hoped that more discussion on this aspect will be provided in the Discussion section.

This is a great point. As mentioned in the Introduction, previous studies have shown that natural *E. coli* promoters often have multiple binding sites in close proximity (DOI: 10.1371/journal.pone.0114347).

We acknowledge and now highlight that there is no definitive threshold for classifying a sequence as a transcription factor binding site. Binding sites exist on a continuum, with affinities ranging from so low as to be negligible, to so high that the transcription factor is nearly always bound (DOI: 10.1038/s41576-024-00713-1). Indeed, binding affinity data for transcription factors, often derived from methods like ChIP-Seq, typically encompass a range of binding peaks rather than a single sequence. These sequences can be affiliated with varying degrees of binding affinity, determined by factors such as experimental enrichment (DOI: 10.1128/microbiolspec.mgm2-0035-2013) or their similarity to a consensus sequence.

In practice, when multiple binding sites with labeled affinities are available for a transcription factor, one can choose among these binding sites as inputs for the SPP method. This flexibility allows for the tailoring of output solutions that meet specific design criteria or assumptions. However, it is important to note that comprehensive binding affinity data is available for only a limited set of transcription factors, and that representative consensus sequences do not necessarily equate to the highest thermodynamic binding affinity (DOIs: 10.1016/S0968-0004(98)01187-6; 10.1186/gb-2003-5-1-201).

We now address some of these nuances in the manuscript and have added text to the Discussion.

→ See Discussion

4. If experimental conditions permit, synthesizing designed promoter sequences and subsequently measuring the strength of artificially designed promoters using methods such as fluorescence protein assays would enhance the persuasiveness of the article.

    We agree that a fluorescent protein assay would be suitable for experimental validation, and we are indeed preparing to screen many SPP-derived promoter sequences using this method. However, as mentioned in the response to Reviewer 1 (page 1 of this document), we believe this falls outside the scope of the current manuscript.

5. The introduction part lacks a comprehensive overview of the categories of computational methods related to promoter design, and there are additional types of computational methods relevant to promoter design that should be introduced. For example, some promoter strength predictive models (classification/regression), which may play a crucial role in in silico directed evolution.

    We thank the reviewer for this comment. We recognize that the field of computational promoter design is broad, covering both predictive and generative methods, each with distinct use cases. The SPP is not related to regression or classification tasks on DNA sequences; it addresses the generative design challenge of creating contiguous DNA sequences from many overlapping binding sites. Since the SPP is inherently generative, our commentary specifically targets other generative methods related to promoter design. We have now expanded our commentary in the Introduction to better explain how the SPP contributes to the generative DNA sequence design space—for example, the SPP can supply synthetic promoters as training examples for deep learning models.

    However, we acknowledge the reviewer's point that SPP-derived promoters could be analyzed using predictive models. These models, typically trained with numerous examples of natural promoters and non-promoter sequences, could then assess our dense array sequences to determine characteristics such as promoter likeness and strength. We now mention in the Discussion how the SPP can be integrated with such predictive models.

    → See Introduction
    → See Discussion

Minor issue: The title of Figure 4 is not bold, inconsistent with other figures.

→ Fixed
